# To explore association between gamma-glutamyl transferase and type 2 diabetes using a real-world study and mendelian randomization analysis

**DOI:** 10.3389/fendo.2022.899008

**Published:** 2022-07-25

**Authors:** Yaru Bi, Shuo Yang, Yanjing Liu, Lingxia Cao, Menghan Gao, Weixia Liu, Yuting Li, Suyan Tian, Chenglin Sun

**Affiliations:** ^1^ Department of Endocrinology and Metabolism, First Hospital of Jilin University, Changchun, China; ^2^ Department of Clinical Nutrition, First Hospital of Jilin University, Changchun, China; ^3^ Department of Internal Medicine, Lvyuan People's Hospital, Changchun, China; ^4^ Division of Clinical Research, First Hospital of Jilin University, Changchun, China

**Keywords:** gamma-glutamyl transferase, type 2 diabetes, epidemiological study, single nucleotide polymorphisms, Mendelian randomization study

## Abstract

**Aim:**

The association between gamma-glutamyl transferase (GGT) and type 2 diabetes mellitus (T2DM) is controversial. In this study, we investigated the association between GGT and the risk of T2DM using real-world data, Mendelian randomization (MR) analysis, and literature mining.

**Methods:**

A cross-sectional study enrolled 3,048 participants (>40 years) from a community in Northeastern China was conducted. A generalized additive model was used to examine the relation between GGT and T2DM. A two-sample MR was performed to investigate the causal effect of GGT (61,089 individuals, mostly of European ancestry) on T2DM (29,193 cases and 182,573 controls of European ancestry).

**Results:**

GGT was related to glucose metabolism indicators, such as fasting plasma glucose and glycosylated hemoglobin (*P* < 0.05). The odds ratios (ORs) [95% confidence interval (95% CI), *P*] for T2DM across the GGT categories (14–16, 17–20, 21–25, 26–35, ≥36) were 1.14 [(0.88-1.47), *P* = 0.330], 1.55 [(1.22-1.98), *P* < 0.001], 1.87 [(1.47-2.28), *P* < 0.001], 1.97 [(1.55-2.52), *P* < 0.001], and 2.29 [(1.78-2.94), *P* < 0.001] versus GGT ≤ 13 category after adjusting for potential confounding factors. A generalized additive model identified a non-linear correlation between GGT and T2DM and indicated that the risk of T2DM almost levelled out when GGT exceeded 34 IU/L. The MR analysis showed that the odds of having T2DM for a one-time increase in genetically determined GGT was 0.998 [(0.995-1.002), *P* = 0.34].

**Conclusions:**

Our analysis of observational study suggested that GGT, its increment, within a certain range, is indicative of the development of T2DM. However, MR analysis provided no evidence that GGT is a linear causal factor of T2DM. Further investigation is required to determine if GGT exerts a non-linear causal effect on T2DM.

## Introduction

The prevalence of diabetes is increasing worldwide, and it is expected to affect up to 700 million people by the year 2045 (1). Type 2 diabetes mellitus (T2DM), which accounts for approximately 90% of diabetic cases, is accompanied by a variety of severe or even fatal complications, such as atherosclerosis, coronary heart diseases, kidney failure, blindness, and limb amputations, making it one of the most important public health challenges (2). Therefore, it is important to identify biochemical markers associated with the risk of T2DM for early detection.

Several studies have investigated the potential biomarkers for early detection of T2DM, such as ferritin, adiponectin and apolipoprotein B (3–5). Compared to these unconventional testing indicators, routine biochemical markers are clinically applicable for the early identification of T2DM. Since the liver plays a major role in the regulation of glucose metabolism, more attention should be paid to commonly used liver function indicators. Several studies have shown that alanine transferase (ALT) and gamma-glutamyl transferase (GGT) are associated with the incidence of T2DM (6–8), and GGT was found to be more related to diabetic morbidities than ALT (9, 10).

Numerous studies have shown that GGT is closely related to metabolic diseases, such as atherosclerosis, coronary heart diseases (11), stroke (12), hypertension (13), and obesity (14). Serum GGT level is widely used as a marker of liver dysfunction and alcoholic consumption. In addition, GGT is involved in the extracellular catabolism of glutathione, an antioxidant, to protect cells against oxidative stress, leading to the conclusion that GGT can also be considered a marker for oxidative stress and inflammation (15, 16). Oxidative stress and inflammation are important pathogenic outcomes of metabolic diseases, including T2DM (17, 18). Therefore, it is proposed that GGT may play essential roles in the pathogenesis of T2DM. However, the relationship between GGT and the risk of diabetes is unclear. Several studies have shown the levels of GGT as a predictor and a risk factor for diabetes (6, 7, 10). In contrast, one study concluded that the GGT level is unassociated with T2DM (19).

Whether the association between GGT and T2DM in observational studies is causal might be addressed by a Mendelian randomization (MR) approach. Specifically, genetic variants (single nucleotide polymorphisms, SNPs) that are strongly associated with the exposure (i.e., GGT) are considered as instrumental variables (IVs) to test the causal effects on the outcome (i.e., T2DM). However, the causal relationship between GGT and T2DM remains controversial. Several MR analyses revealed that genetically elevated GGT levels had no causal effect on T2DM (20, 21). On the contrary, Lee et al. (2016) showed that GGT levels may have a causal role in the development of T2DM (22).

As mentioned above, whether GGT is associated with T2DM morbidity and whether the association is causal are both controversial. It is worthy pointing out that previous studies were either observational or carried out MR analysis to investigate the relationship between GGT and T2DM. In this study, we conducted a comprehensive analysis that integrated a large-sized cross-sectional study and an MR study to provide a unified answer to these two controversial issues. In addition to this, the current study has other major advantages. First, unlike previous MR studies, a newly released genome-wide association study (GWAS) with a larger sample size was used as the source of T2DM, which offers more statistical power to detect a subtle effect. Second, for the observational study, given that a few studies specifically focused on the Chinese population to determine the relationship between GGT and T2DM, especially the potential dose-response relationship between GGT and T2DM, our study is of the first one that fulfills this gap.

## Material and methods

### Study population

We recruited 3,325 participants, aged ≥40 years, who participated in the conventional physical examinations in Community Puyang in Changchun City, a northeastern city in Jilin Province, from January 2019 to January 2021. The exclusion criteria were as follows: type 1 diabetes, gestational diabetes, and other types of diabetes; self-reported history of liver and/or kidney diseases; acute cardiovascular diseases, stroke, acute infection, severe trauma, stress, surgery, etc. in the recent 3 months; a history of anemia; recent use of corticosteroid, excessive alcohol consumption (>30g/d in men or >20 g/d in women). We also excluded the participants with missing data. After removing the missing data, 3048 individuals were eventually enrolled, including 955 men and 2,093 women.

### Data collection and measurements

Each participant received a physical examination that involved measurements of height, weight, and blood pressure and completed a questionnaire that included their personal information, lifestyle, and self-reported medical history. After overnight fasting of 8-12 h, the venous blood sample was collected to measure the levels of fasting plasma glucose (FPG), ALT, aspartate transaminase (AST), GGT, glycosylated hemoglobin (HbA1c), triglyceride (TG), and total cholesterol (TC) [low-density lipoprotein cholesterol (LDL-C) and high-density lipoprotein cholesterol (HDL-C)].

Based on their alcohol consumption, patients were divided into three categories: non-drinkers, occasional drinkers (not weekly), and heavy drinkers (weekly or almost weekly). Alcohol drinking was evaluated by the type and amount of alcoholic beverage consumed per week. The participants were also classified into three groups based on their smoking status: non-smokers, occasional smokers (less than one per day or less than 7 cigarettes per week), and heavy smokers (daily or almost daily use of cigarettes).

According to the American Diabetes Association, T2DM is diagnosed when the participants meet any one of the following criteria: FPG ≥7.0 mmol/L, and/or HbA1c ≥6.5%, and/or report of a doctor’s diagnosis or taking hypoglycemic drugs. Prediabetes is defined when the HbA1c level ranges between 5.7% and 6.5%, and hypertension is defined when systolic blood pressure ≥140mmHg and/or diastolic blood pressure ≥90mmHg. Body Mass Index (BMI) is calculated as weight (kg) divided by the square of height (m^2^).

### Statistical analyses of the observational study

Data were expressed as mean ± standard deviation (SD), or median (interquartile) for continuous variables, if appropriate, and as a percentage for categorical variables. The statistical differences between normoglycemia, prediabetes and T2DM group, also among sextiles of GGT were detected by performing the analysis of variance (for normally distributed variables) and Kruskal-Wallis tests (for variables with a skewed distribution) for continuous variables and chi-square tests for categorical variables. Odds ratios (ORs) and their corresponding 95% confidence intervals (CIs) utilizing multiple logistic regression were calculated to examine the relationship between GGT levels and the risk of T2DM. Adjustments for variables in the logistic regression were used to control the confounding factors and mitigate their impact in assessing the relationship between GGT and T2DM. Model 1 was adjusted for basal variables of gender and age. Model 2 was further adjusted for hypertension, BMI, and smoking and drinking status, while model 3 additionally was adjusted for potential biochemical indicators including TG and ALT. A generalized additive model was fit to evaluate the relationship between GGT actual values and the risk of T2DM, and a partial dependence plot was drawn to present the results. *P* < 0.05 was considered statistically significant. Statistical analyses were performed using SPSS (version 22.0; IBM Corporation, Armonk, NY, USA).

### Mendelian randomization

Two-sample MR analysis was performed to understand if the relationship between GGT and T2DM is causal, in which GGT is considered as the exposure and T2DM is the outcome of interest using the MR-Base online platform (available at https://app.mrbase.org) (23). Specifically, we selected multiple independent SNPs (low linkage disequilibrium *R^2^* < 0.001) associated with GGT at genome-wide significance (*P* < 5×10^-8^) from Chambers et al., including 61,089 individuals (52,350 individuals of European ancestry and 8,739 individuals of Indian Asian ancestry) from a 21 population-based cohort (24). For T2DM, we used data from the FinnGen consortium comprising 29,193 cases and 182,573 controls of European ancestry. A proxy SNPs (R^2^ > 0.8) was automatically conducted by MR-Base.

In the MR analysis, the conventional inverse-variance weighted (IVW) method, the MR-Egger regression method, the weighted median estimator, and the weighted mode method were implemented to evaluate the causal relationship between GGT and T2DM. Furthermore, the intercept of MR-Egger, funnel plots, and Cochrane’s Q tests were used to assess the horizontal pleiotropy and heterogeneity. Multiplicative random effects models were used when SNPs exhibited heterogeneity. Lastly, the MR Steiger filtering was conducted to test whether individual SNPs are GGT-related or T2DM-related, thus inferring the causal direction (to eliminate the reverse causality).

The statistical power of the MR analysis was calculated using an online calculator, i.e., mRnd (https://cnsgenomics.com/shiny/mRnd/). The Steiger filtering was carried out in R software 4.1.0 (http://www.r-project.org/) using the R package “TwoSampleMR”.

The paper was prepared with reference to the STROBE and STROBE-MR statements for the cross-sectional study and MR study. The two checklists were presented in the **Supplementary Files 1** and **2**.

## Results

In this real-world evidence study, 3,325 participants were recruited, and 277 individuals were excluded due to data missing. Finally, 3,048 participants, having a mean age of 57.0 ± 9.2 years, were included, of which 68.7% (2093/3048) were females. The baseline characteristics of all subjects as per their blood glucose levels are listed in **Table 1**. T2DM group consisted of significantly older patients with higher BMI, TG, ALT, GGT, and a large proportion of them suffering from hypertension when compared to the normoglycemia and the prediabetes groups, whereas HDL-C levels showed an opposite trend (*P*<0.05).

**Table 1:** Characteristics of the study population by categories of blood glucose.

Variables	Normoglycemia group (n=1026)	Prediabetes group (n=1223)	T2DM group (n=799)	*P*
Female (%)	678 (66.1)	887 (72.5)	528 (66.1) ^‡^	<0.001
Age (years)	53.4 ± 9.3	58.1 ± 8.7	59.8 ± 8.2 ^†,‡^	<0.001
Hypertension (%)	450 (43.9)	603 (49.3)	496 (62.1) ^†,‡^	<0.001
BMI (kg/m^2^)	24.5 ± 3.0	25.0 ± 3.0	25.8 ± 2.9 ^†,‡^	<0.001
FPG (mmol/L)	5.3 ± 0.5	5.6 ± 0.5	8.0 ± 2.3 ^†,‡^	<0.001
HbA1c (%)	5.4 ± 0.2	6.0 ± 0.2	7.2 ± 1.3 ^†,‡^	<0.001
TC (mmol/L)	5.0 ± 0.9	5.2 ± 0.9	5.3 ± 1.0 ^†^	<0.001
TG (mmol/L)	1.5 ± 1.1	1.8 ± 1.2	2.1 ± 1.5 ^†,‡^	<0.001
LDL-C (mmol/L)	2.8 ± 0.7	3.0 ± 0.7	3.0 ± 0.8 ^†^	<0.001
HDL-C (mmol/L)	1.3 ± 0.3	1.3 ± 0.3	1.2 ± 0.3 ^†,‡^	<0.001
ALT (IU/L)	13.0 ± 6.1	12.6 ± 6.2	14.3 ± 6.7 ^†,‡^	<0.001
AST (IU/L)	20.4 ± 5.1	20.1 ± 4.8	20.2 ± 5.5	0.026
GGT (IU/L)	26.1 ± 20.9	25.7 ± 19.2	30.4 ± 19.5 ^†,‡^	<0.001
Smoking status
Nonsmoker, N (%)	854 (83.2)	1052 (86.0)	688 (86.1)	0.053
Occasional smoker, N (%)	23 (2.2)	27 (2.2)	17 (2.1)	
Heavy smoker, N (%)	149 (14.5)	144 (11.8)	94 (11.8)	
Drinking status
Nondrinker, N (%)	773 (75.3)	983 (80.4)	641 (80.2) ^†^	<0.001
Occasional drinker, N (%)	161 (15.7)	150 (12.3)	101 (12.6) ^†^	
Heavy drinker, N (%)	92 (9.0)	90 (7.3)	57 (7.1)	

BMI, body mass index; FPG, fasting plasma glucose; HbA1c, hemoglobin A1c; TC, total cholesterol; TG, triglyceride; LDL-C, low-density lipoprotein; HDL-C, high-density lipoprotein; ALT, alanine aminotransferase; AST, aspartate aminotransferase; GGT, gamma-glutamyl transferase.

Data are expressed as means ± standard deviations or median (interquartile ranges) for continuous variables and number (percentage) for categorical variables.

^†^T2DM group is statistically significant compared to normoglycemia with P<0.05.

^‡^T2DM group is statistically significant compared to prediabetes group with P<0.05.

The characteristics of the study population according to the sextiles of GGT levels are shown in **Table 2**. The proportion of hypertension was larger, the levels of BMI, FPG, HbA1c, TC, TG, ALT, AST, and LDL-C were higher, and the level of HDL-C was lower when the GGT levels increased (*P* < 0.05). For example, HbA1c increased from 5.8 ± 0.7% in the first sextile to 6.2 ± 1.1% in the fifth sextile, the proportion of T2DM also increased from 13.8% in the lowest sextile to 32.0% in the fifth sextile, and the differences were statistically significant (*P*<0.05). On the other hand, FPG, HbA1c, and the proportion of T2DM in the sixth sextile group were not statistically significant compared to the fifth sextile group (*P* > 0.05).

**Table 2:** Clinical and demographic characteristics of the study population stratified by the sixtiles of GGT levels.

Variables	GGT						
	S1 (≤13)	S2 (14-16)	S3 (17-20)	S4 (21-25)	S5 (26-35)	S6 (≥36)	*P*
Participants	485	462	501	486	534	580	
GGT (IU/L)	12 (11-13)	15 (14-16)	18 (17-19)	23 (22-24)	29 (27-32)	49 (41-66)	<0.001
Females (%)	429 (88.5)	365 (79.0)	372 (74.2)	324 (66.7)	314 (58.8)	289 (49.8)	<0.001
Age (years)	55.3 ± 9.8	57.2 ± 9.5	58.6 ± 9.2	57.6 ± 8.9	57.3 ± 8.6	55.9 ± 8.7	<0.001
Hypertension (%)	172 (35.5)	200 (43.3)	263 (52.5)	264 (54.3)	300 (56.2)	350 (60.3)	<0.001
BMI (Kg/m2)	23.7 ± 2.8	24.1 ± 2.9	25.0 ± 2.9	25.4 ± 2.8	25.7 ± 3.0	26.0 ± 3.0	<0.001
FPG (mmol/l)	5.6 ± 1.1	5.8 ± 1.4	6.1 ± 1.5	6.2 ± 1.7	6.4 ± 1.9^†^	6.6 ± 2.0^‡^	<0.001
HbA1c (%)	5.8 ± 0.7	5.9 ± 0.8	6.1 ± 0.9	6.2 ± 1.0	6.2 ± 1.1^†^	6.3 ± 1.2 ^‡^	<0.001
TC (mmol/l)	4.8 ± 0.9	5.0 ± 0.9	5.1 ± 0.9	5.1 ± 0.9	5.2 ± 0.9	5.4 ± 1.0	<0.001
TG (mmol/l)	1.2 ± 0.6	1.5 ± 1.1	1.6 ± 1.0	1.8 ± 1.3	2.1 ± 1.4	2.3 ± 1.7	<0.001
LDL-C (mmol/l)	2.7 ± 0.7	2.9 ± 0.7	2.9 ± 0.7	3.0 ± 0.8	3.0 ± 0.8	3.1 ± 0.8	<0.001
HDL-C (mmol/l)	1.4 ± 0.3	1.3 ± 0.3	1.3 ± 0.3	1.3 ± 0.3	1.3 ± 0.3	1.3 ± 0.3	<0.001
ALT(IU/L)	9 (7-12)	10 (8-14)	11 (8-15)	12 (9-16)	14 (10-19)	17 (12-23)	<0.001
AST(IU/L)	18 (15-20)	18 (16-21)	19 (16-21)	19 (17-22)	20 (17-24)	22 (19-26)	<0.001
Glycemia status							
Normoglycemia, N (%)	215 (44.3)	180 (39.0)	158 (31.5)	145 (29.8)	146 (27.3)	182 (31.4)	<0.001
Prediabetes, N (%)	203 (41.9)	199 (43.1)	214 (42.7)	198 (40.7)	217 (40.6)	192 (33.1)	
T2DM, N (%)	67 (13.8)	83 (18.0)	129 (25.7)	143 (29.4)	171 (32.0) ^†^	206 (35.5) ^‡^	

^†^S5 is statistically significant compared to S1 with P < 0.05.

^‡^S6 is not statistically significant compared to S5 with P>0.05.

The ORs and 95% CIs for the risk of T2DM determined by serum GGT levels are shown in **Table 3**. In the crude model, the ORs [(95% CIs), *P*] of T2DM were 1.37 [(1.07–1.75), *P* = 0.014] in sextile 2; 2.16 [(1.71–2.72), *P*<0.001] in sextile 3; 2.62 [(2.08–3.29), *P*<0.001] in sextile 4; 2.92 [(2.34–3.65), *P*<0.001] in sextile 5 and 3.42 [(2.75–4.25), *P*<0.001] in sextile 6, compared to GGT sextile 1 (considered as reference here). This association was evident even after potential confounders gender, age, BMI, hypertension, smoking status, and alcohol drinking adjustments. Further additional adjustments for TG and ALT revealed an approximately 2.3-fold higher risk of developing T2DM (OR = 2.29, 95% CI: 1.78–2.94, *P*<0.001) in participants categorized in the highest sextile (concerning GGT levels) compared to the lowest sextile.

**Table 3:** Multiple logistic regression models examining the association between serum GGT level and T2DM.

GGT(IU/L)	Crude	Model 1	Model 2	Model 3
S1(≤13)	1	1	1	1
S2(14-16)	1.37 (1.07-1.75)[*P* = 0.014]	1.28 (0.99-1.65) [*P* = 0.054]	1.23 (0.95-1.59) [*P* = 0.110]	1.14 (0.88-1.47) [*P* = 0.330]
S3(17-20)	2.16 (1.71-2.72)[*P*<0.001]	1.94 (1.53-2.46) [*P*<0.001]	1.74 (1.37-2.20) [*P*<0.001]	1.55 (1.22-1.98) [*P*<0.001]
S4(21-25)	2.62 (2.08-3.29) [*P*<0.001]	2.52 (1.99-3.18) [*P*<0.001]	2.18 (1.72-2.76) [*P*<0.001]	1.87 (1.47-2.28) [*P*<0.001]
S5(26-35)	2.92 (2.34-3.65) [*P*<0.001]	2.90 (2.30-3.65) [*P*<0.001]	2.45 (1.94-3.10) [*P*<0.001]	1.97 (1.55-2.52) [*P*<0.001]
S6(≥36)	3.42 (2.75-4.25) [*P*<0.001]	3.71 (2.95-4.65) [*P*<0.001]	3.10 (2.45-3.92) [*P*<0.001]	2.29 (1.78-2.94) [*P*<0.001]

Model 1: adjusting for gender and age.

Model 2: adjusting for all factors in model 1+ hypertension, BMI, alcohol drinking, smoking status.

Model 3: adjusting for all factors in model 2+ TG and ALT.

**Figure 1:**
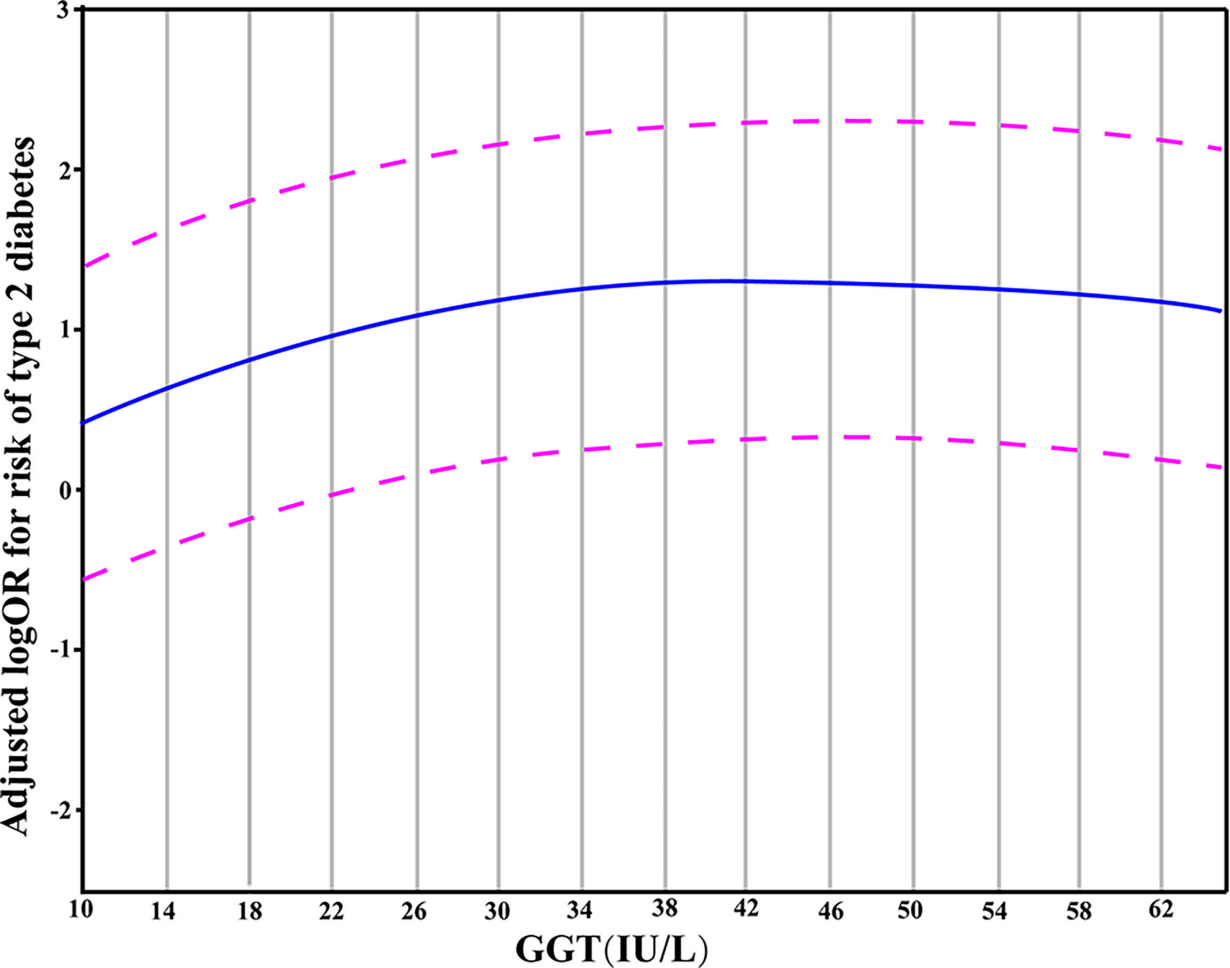
A partial dependence plot given by the generalized additive model in which the adjustment for gender, age, hypertension, BMI, smoking, alcohol drinking, TG and ALT was made. The blue line represented the estimated OR of incident T2DM, and the purple dotted line indicated 95% CI.

The results from the generalized additive model indicated that the relationship between GGT levels and T2DM is non-linear. Specifically, a GGT level of 34 IU/L corresponded to the peak risk of developing T2DM. In this model, the confounding effects of gender, age, smoking, alcohol drinking, hypertension, BMI, TG, and ALT values were adjusted (**Figure 1**).

Next, we explored if a causal relationship existed between GGT levels and T2DM by carrying out a two-sample MR analysis, in which the genetic variants of GGT were taken as IVs. At the significance level of 0.05, a sample size of 211,766 (the sample size of FinnGen study) had a power of>0.9 to detect a causal risk of 1.1 to develop T2DM per standard deviation increase in GGT level (assuming the genetic variants explain away 5% of the variance in GGT).

**Figure 2:**
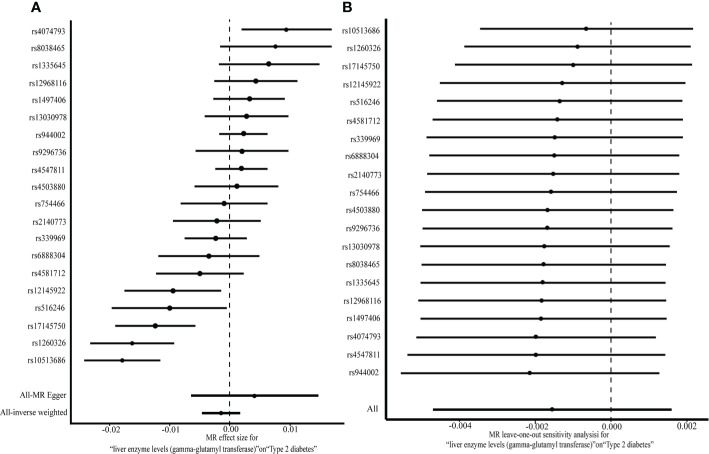
A two-sample MR analysis of the effect of GGT on T2DM **(A)** and MR leave-one-out analysis for GGT on T2DM **(B)**.

No pleiotropic effects (the intercept of MR-Egger regression was –0.023, *P* = 0.297) of SNPs on the outcome were found. Since the SNPs were heterogeneous according to heterogeneity statistics (Cochran’s Q value = 84.56, *P* = 1.349e-10 for MR Egger, and Q value = 89.98, *P* = 3.339e-11 for the IVW method) and the funnel plot, the multiplicative random effects of IVW were fit. No causal association between GGT (one-time increase) and T2DM was found based on the genetic score of 20 GGT-related SNPs, by using the random effects IVW model (OR [95% CI]:0.998 [0.995,1.002], *P* = 0.34) or the MR-Egger (1.004 [0.993,1.015], *P* = 0.47) (**Figure 2A**). Additionally, the same null results were observed with the weighted median (1.002 [0.999,1.004], *P* = 0.16) and the weighted mode (1.002 [0.999,1.004], *P* = 0.18). In a leave-one-out sensitivity analysis, no SNPs that significantly influenced the causal association of GGT with T2DM were detected (**Figure 2B** **)**. The Steiger filtering method verified the direction of the causality should be from GGT to T2DM.

Lastly, we carried out a literature search on previously conducted MR studies and summarized the results in **Table 4** (20–22, 25, 26). Except for the study that used Korean data by Lee et al. (2016), which revealed a weak genetic connection between GGT levels and the development of T2DM, others indicated no causal relation between GGT and T2DM.

**Table 4:** Summary of the Mendelian randomization study verifying the causal relationship of GGT on T2DM.

Lead author, publication year	Number of SNP	GGT-related variants	T2DM related variants	MR-IVW	The unit of GGT
Silva, 2019	26	GWAS	GWAS DIAGRAM	0.92 (0.80-1.06)	1SD
Nano, 2017	26	GWAS	DIAGRAM	0.898 (0.759-1.090)	1SD/logGGT
Liu, 2016	24	GWAS	DIAGRAM	0.88 (0.75-1.04)	two times
Lee, 2016 (Two sample MR method)	7	GWAS	GWAS	1.007 (0.993-1.022)	1 unit
Lee, 2016 (Korean data)	7	GWAS	GWAS	1.024 (1.001-1.048)	1 unit
Noordam, 2016	26	GWAS	DIAGRAM	0.998 (0.954-1.024)	10% higher

SNPs, single nucleotide polymorphisms; GWAS, Genome-wide association study; MR-IVW, Mendelian randomization inverse-variance weighted.

## Discussion

In our study, we observed that GGT levels in the T2DM group were higher than that in the normoglycemia and prediabetes groups. It was also seen that glucose metabolism indicators also increased with the increase in GGT levels. For example, the level of HbA1c increased from 5.8% in the first sextile to 6.2% in the fifth sextile, and accordingly proportion of T2DM patients increased from 13.8% to 32.0%, respectively, and these differences were statistically significant. This is consistent with the results of a cross-sectional study conducted by Sabanaygam et al. (2009) (27), in which the participants over 20-year-old were enrolled in the United States; the HbA1c level and the proportion of diabetics increased with the rise in GGT levels. Besides, the cross-sectional analysis, based on the DRYAD dataset, also revealed a similar pattern (28). Further analysis showed that glucose metabolism biomarkers such as FPG, HbA1c, and the proportion of occurrence of T2DM were not varying statistically between the fifth sextile and the sixth sextile, which suggested the correlation between GGT levels and the risk of developing T2DM existed only till a certain threshold point.

The biological mechanisms underlying the association between the GGT levels and the development of T2DM might be explained by oxidative stress and inflammation. Serum GGT levels increase to maintain the intracellular antioxidant glutathione. Therefore, GGT is closely related to oxidative stress. For example, experimental studies found that GGT protected cells from oxidation-induced cell death (29), and GGT deficiency caused an increase in oxidative stress (30). Epidemiological studies found that serum antioxidants (carotene and vitamin C) in the body were inversely associated with serum GGT levels within its normal range (31). The oxidative stress might damage DNA, proteins, and lipids, which can further activate oxidative stress-related pathways, that might damage the structure and function of β-cells of pancreatic islets (32). Furthermore, oxidative stress potentiates the generation of various inflammatory cytokines such as tumor necrosis factor-α and interleukin-6. Low-grade chronic inflammation might play a role in the pathogenesis of diabetes (18). Together low insulin secretion and inflammation can promote the occurrence of T2DM, indicating that the risk of T2DM only increases within a certain range of GGT.

Next, using the actual values of GGT, a generalized additive model was fit to explore more deeply the association pattern between GGT and T2DM. The results showed that the risk of T2DM did not increase when the GGT levels exceeded 34 IU/L. In other words, there may be a saturation effect between them. A meta-analysis of 16 studies provided a consistent result and illustrated a saturation effect between GGT and diabetes with the corresponding GGT level of 35 IU/L (33). We thus propose a hypothesis to explain the non-linear relationship between GGT and T2DM: the oxidative stress in the body rises during the early onset of diabetes, and the levels of GGT (with its anti-oxidative ability) increase to a certain level to resist the oxidative stress caused by the diabetes condition. However, with the progression of diabetes, the antioxidant activity of GGT is insufficient to overcome the oxidative stress caused by T2DM. Consequently, the GGT level does not influence or become a risk factor for T2DM development once it exceeds a certain threshold value (34 IU/L). However, further investigations are warranted to test this hypothesis.

MR analysis generally uses genetic variants (SNPs) as IVs to investigate the causal relationship between the two variables. We applied a two-sample MR analysis to explore the causal relationship between GGT and T2DM using GGT as exposure and T2DM as outcomes. The results showed that GGT levels might have no causal role in the development of T2DM. A literature search on the Mendelian randomization analysis is summarized in **Table 4**. Initially, we planned to carry out a meta-analysis using these five studies (20–22, 25, 26). Because of two reasons, such an analysis was discarded. First, there were huge differences in the units used for assessing GGT values across different studies, and thus, it became difficult to summarize the five studies and to estimate the causal effect of GGT on T2DM. Second, we found that the data considered in these studies overlapped substantially. It is inappropriate to conduct a meta-analysis using largely overlapping data to estimate the causal effect of GGT on T2DM. However, none of the previous MR studies, except for one (22), showed any causal association. Thus, we concluded that GGT might have no causal impact on T2DM. Notably, our observations and analysis suggest a non-linear correlation between GGT and T2DM; however, a linearity assumption was made in MR studies. Hence, the results from the MR study should be interpreted with caution.

The cross-sectional study (with extensive data obtained from over 3000 participants) indicates a non-linear association between GGT and T2DM; the relationship between them may not be linear causal as per MR analysis and literature mining. Our study made several contributions to the field. First, we integrated an observational study, MR analysis, and literature mining together to investigate the relationship between GGT and T2DM, which presents the clinicians with a new research strategy, namely, combining an observational study and MR study to deeply explore the relationship between two attributes. Our results were more comprehensive compared to those that were based only on an MR study or an observational study. Second, for the observational study, to our knowledge, this study is a pioneer in specifically focusing on the Chinese population to explore a possible non-linear correlation between GGT and T2DM. Also, we reasoned from the perspective of oxidative stress to explain this non-linear relationship and determined an explicit cutoff value of GGT 34IU/L at which the correlation between GGT and T2DM disappears. Third, for the two-sample MR analysis, previous MR study mainly used DIAGRAM as the source of T2DM, we used FinnGen study, a larger cohort study than DIAGRAM and has not been used in previous MR studies, as the source of T2DM instead.

This study had several limitations. First, undiagnosed hepatic conditions, such as nonalcoholic fatty liver disease, might be confounding factors for the association between GGT and T2DM, and we could get rigorous results if the model adjusted for hepatic conditions. Nevertheless, in the community-based observational study, the participants did not undergo ultrasound or hematological examinations for the formal diagnosis of hepatic conditions. However, the individuals with self-reported hepatic diseases were excluded when the participants were enrolled. Consequently, the proportion of undiagnosed hepatic conditions in the current study was not high. Thus, we argued that the association between GGT and T2DM might not be significantly altered considering the large-sized clinical data. Second, in a two-sample MR analysis, for the exposure (GGT), participants had a mixed ancestry (85.7% of European ancestry and 14.3% of Indian Asian ancestry), while participants of the outcome (T2DM) were from the FinnGen study. The Finnish population is genetically different from conventional European populations in terms of linkage disequilibrium structure and allele frequencies. The genetic differences would lead to a bias in the estimation of the causal effect. Third, most GGT-related SNPs and diabetes-related SNPs (**Table 4**) were from European ancestry genome-wide association studies, and similar data on the Chinese population are not available. Last, the participants included in the real-world evidence study were from the northeast area of China, which may not represent the characteristics of the general Chinese population.

In conclusion, the findings of the observational study indicated that GGT is non-linearly correlated to the risk of T2DM. The MR analysis suggested that GGT is not a linear causal risk factor for T2DM. However, we could not exclude the possibility of GGT having a non-linear causal effect on T2DM. In the future, we will adopt the inverse propensity weighting method and the restricted cubic spline method to evaluate if such a non-linear causality exists between GGT and T2DM.

## Data Availability Statement

The original contributions presented in the study are included in the article/supplementary material. Further inquiries can be directed to the corresponding authors.

## Ethics Statement

The studies involving human participants were reviewed and approved by Ethics Committee of the First Hospital of Jilin University. The patients/participants provided their written informed consent to participate in this study.

## Author Contributions

CS and ST contributed to conception and design of the study. YB, SY, YL were responsible for writing. LC, MG, WL, YL were responsible for the data collection. YB and ST performed the statistical analysis. All authors contributed to manuscript revision, read, and approved the submitted version.

## Funding

This work was supported by the Science Technology Department of Jilin Province (20200404213YY).

## Acknowledgments

We would like to thank the subjects for their support and participation in the study. We want to acknowledge the participants and investigators of FinnGen study.

## Conflict of Interest

The authors declare that the research was conducted in the absence of any commercial or financial relationship that could be constructed as a potential conflict of interest.

## Publisher’s Note

All claims expressed in this article are solely those of the authors and do not necessarily represent those of their affiliated organizations, or those of the publisher, the editors and the reviewers. Any product that may be evaluated in this article, or claim that may be made by its manufacturer, is not guaranteed or endorsed by the publisher.
